# Factors Associated with Documented Arrhythmia Recurrence After Cryoballoon Ablation in a Low-Risk Atrial Fibrillation Population Without Major Comorbidities

**DOI:** 10.3390/jcm15166448

**Published:** 2026-08-20

**Authors:** Murat Erdem Alp, Veli Polat, Süleyman Barutçu, Güngör İlayda Bostancı Alp, Yaser İslamoğlu, Gazi Çapar, Eyüp Özkan, Taylan Akgün

**Affiliations:** Department of Cardiology, Başakşehir Çam and Sakura City Hospital, 34480 İstanbul, Turkey; dr.velipolat@gmail.com (V.P.); slybrtcu@gmail.com (S.B.); gilaydabostanci@hotmail.com (G.İ.B.A.); yaserislamoglu@gmail.com (Y.İ.); gazicapar1@gmail.com (G.Ç.); dreyupozkan@gmail.com (E.Ö.); taylanakgun@gmail.com (T.A.)

**Keywords:** atrial fibrillation, cryoballoon ablation, arrhythmia recurrence, female sex, CHA_2_DS_2_-VA score

## Abstract

**Background:** Arrhythmia recurrence after cryoballoon ablation remains a clinically relevant problem in atrial fibrillation (AF). However, factors associated with documented recurrence in low-risk patients without major comorbidities are not well defined. This study aimed to evaluate factors associated with documented arrhythmia recurrence after cryoballoon ablation in a highly selected low-risk AF population. **Methods:** This retrospective, single-center study included 153 eligible patients selected from an institutional cryoablation database after application of predefined exclusion criteria. Only patients with a CHA_2_DS_2_-VA (congestive heart failure, hypertension, age ≥ 75 years, diabetes mellitus, stroke/transient ischemic attack/thromboembolism, vascular disease, and age 65–74 years) score ≤ 1 and without major comorbidities, including diabetes mellitus, hypertension, coronary artery disease, chronic kidney disease, cerebrovascular disease, and heart failure, were included. Recurrence was defined as electrocardiographically documented AF or atrial tachyarrhythmia after the 3-month blanking period over 1 year of follow-up; Holter-detected episodes were required to last ≥30 s. The primary multivariable model included sex, age, AF type, and absolute left atrial diameter; a sensitivity model replaced absolute left atrial diameter with left atrial diameter indexed to body surface area (BSA). **Results:** The study population included 70 women (45.8%) and 83 men (54.2%). Paroxysmal AF was present in 125 patients (81.7%), whereas 28 patients (18.3%) had persistent AF. Documented arrhythmia recurrence occurred in 40 patients (26.1%). Female sex was more frequent in the recurrence group than in the no-recurrence group (62.5% vs. 39.8%, *p* = 0.013). The complete-case primary multivariable model included 126 patients with 36 recurrence events. Female sex was associated with documented arrhythmia recurrence (odds ratio [OR] 2.69, 95% confidence interval [CI] 1.14–6.37; *p* = 0.024). The estimate for left atrial diameter was directionally positive but statistically uncertain (OR 1.086 per mm, 95% CI 0.989–1.192; *p* = 0.085). In the BSA-indexed sensitivity model (n = 123), the female-sex estimate was attenuated and statistically uncertain (OR 2.16, 95% CI 0.90–5.18; *p* = 0.084), while left atrial diameter/BSA was also statistically uncertain (OR 1.145 per mm/m^2^, 95% CI 0.977–1.343; *p* = 0.095). **Conclusions:** In this selected low-risk cohort undergoing second-generation cryoballoon ablation, female sex was associated with clinically detected, electrocardiographically documented arrhythmia recurrence in the primary model under an intermittent rhythm-surveillance strategy based on scheduled 12-lead electrocardiograms (ECGs) and symptom-driven evaluations. However, the estimate was attenuated and statistically uncertain after indexing left atrial diameter to BSA. Given the retrospective, single-center design, non-systematic rhythm monitoring, and sensitivity of the sex estimate to body-size adjustment, these findings should be interpreted as hypothesis-generating.

## 1. Introduction

Atrial fibrillation (AF) is the most common sustained cardiac arrhythmia and is associated with increased risks of heart failure, stroke, impaired quality of life, and mortality [1,2,3,4,5]. Catheter ablation has become an established rhythm-control strategy in symptomatic patients with AF, and cryoballoon ablation is widely used because of its procedural standardization, reproducibility, and favorable learning curve.

Despite the effectiveness of catheter ablation, arrhythmia recurrence remains a significant clinical challenge. Numerous studies have identified several predictors of recurrence after AF ablation, including left atrial enlargement, persistent AF, age, sex, and various laboratory parameters reflecting systemic inflammation or disease burden [6,7,8,9,10]. In addition, clinical risk scores such as CHA_2_DS_2_-VASc have been reported to correlate with recurrence, probably because they reflect the cumulative impact of cardiovascular comorbidities and systemic risk factors [11,12,13].

However, most of the available evidence has been derived from heterogeneous AF populations with a relatively high burden of cardiovascular comorbidities and elevated CHA_2_DS_2_-VASc scores. In such populations, recurrence after ablation may be driven not only by atrial substrate and procedural factors, but also by systemic disease burden, inflammation, and comorbidity-related atrial remodeling. Therefore, predictors identified in these cohorts may not be directly applicable to patients with very low clinical risk.

In contrast, catheter ablation is increasingly performed at earlier stages of AF, resulting in a growing population of younger patients with fewer comorbidities and lower thromboembolic risk scores undergoing ablation. In this selected low-risk population, the mechanisms underlying recurrence may differ, and the relative contribution of sex, AF type, left atrial remodeling, renal function, body mass index, and routine inflammatory markers remains insufficiently defined.

According to the 2024 ESC guidelines, the CHA_2_DS_2_-VA score, in which the sex category is removed from the CHA_2_DS_2_-VASc score, is recommended for thromboembolic risk stratification in patients with AF. In the present study, we investigated factors associated with arrhythmia recurrence over 1 year of follow-up after cryoballoon ablation in a highly selected low-risk AF population, defined by a CHA_2_DS_2_-VA score ≤ 1 and the absence of major comorbidities, including diabetes mellitus, hypertension, coronary artery disease, chronic kidney disease, cerebrovascular disease, and heart failure. We hypothesized that female sex and established substrate-related variables, particularly AF type and left atrial size, could remain associated with documented recurrence even in patients with a low conventional comorbidity burden.

## 2. Methods

### 2.1. Study Population

**Figure 1:**
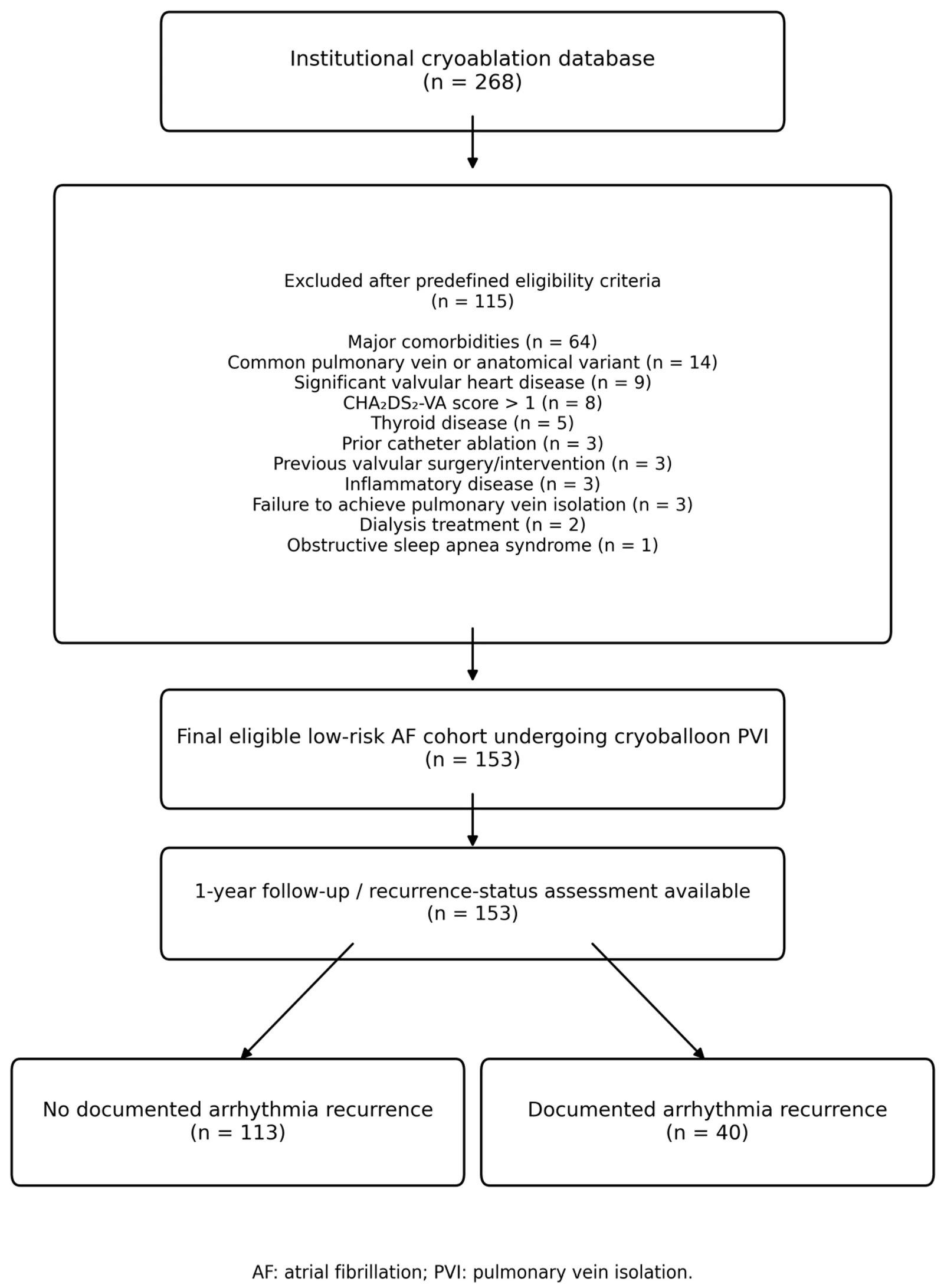
Patient-selection flow diagram with exclusion counts and 1-year follow-up outcome status.

The institutional cryoablation database was retrospectively reviewed and included 268 patients who underwent cryoablation procedures during the study period. After application of the predefined exclusion criteria and patient-level verification of eligibility, 115 patients were excluded and 153 patients who underwent cryoballoon ablation for pulmonary vein isolation due to atrial fibrillation between August 2020 and July 2024 were included in the final analysis. The reasons for exclusion and the follow-up outcome status of the included cohort are summarized in Figure 1. Recurrence-status assessment through 1 year was available for all 153 included patients.

AF type was classified according to guideline-based temporal criteria [1]. Paroxysmal AF was defined as AF that terminated spontaneously or with intervention within 7 days of onset, whereas persistent AF was defined as AF continuously sustained beyond 7 days, including episodes terminated by cardioversion after ≥7 days.

Baseline laboratory measurements and transthoracic echocardiography were obtained within 24 h before ablation. Left atrial diameter was measured as the anteroposterior dimension in the parasternal long-axis view at end-systole, using the routine echocardiographic measurement protocol. Values were extracted from the formal echocardiography reports and were not independently re-measured for the purposes of this retrospective study. Body surface area was calculated from the available height and weight data, and a body-size-indexed left atrial measure was derived by dividing left atrial diameter (mm) by BSA (m^2^), expressed as mm/m^2^.

Patients were excluded if they met any of the following criteria:

- History of hypo- or hyperthyroidism (n = 5).
- Prior catheter ablation (n = 3).
- Age < 18 years.
- Previous valvular surgery or intervention (n = 3).
- Significant valvular heart disease (n = 9).
- Inflammatory diseases (n = 3).
- Obstructive sleep apnea syndrome (OSAS) (n = 1).
- Failure to achieve pulmonary vein isolation (n = 3).
- Presence of a common pulmonary vein or a pulmonary venous anatomical variant (n = 14).
- Receiving dialysis treatment (n = 2).
- Major comorbidities, including diabetes mellitus, hypertension, coronary artery disease, chronic kidney disease, cerebrovascular disease, or heart failure (n = 64).
- CHA_2_DS_2_-VA score > 1 (n = 8).

### 2.2. Ablation Procedure

Cryoballoon ablation was performed with a second-generation 28-mm cryoballoon catheter in all patients. Procedures were performed under conscious sedation using a light-to-moderate sedation strategy.

Pulmonary vein isolation was achieved in all patients. No fixed universal freeze duration was used; freeze-application duration was individualized according to the electrical response of the pulmonary vein and the achieved cryoballoon temperature. When pulmonary vein potentials were recordable during the application, real-time disappearance of the pulmonary vein signal was used to guide the application; however, exact time-to-isolation values were not systematically stored in the retrospective database. A nadir temperature of approximately −55 °C was used as a procedural cryothermal target when accompanied by electrical isolation. Additional freeze applications were delivered when pulmonary vein potentials persisted or when entrance and exit block could not be demonstrated after the initial application. Pulmonary vein isolation was confirmed by demonstrating both entrance and exit block. Patients in whom durable electrical isolation could not be achieved were excluded from the study. No standardized dedicated phrenic-nerve monitoring protocol (such as systematic diaphragmatic pacing with compound motor action potential monitoring) was used; consequently, formal phrenic-monitoring data were not available for retrospective analysis. All procedures were performed by experienced operators.

Procedural parameters extracted from the procedural reports included pulmonary vein-specific nadir temperature and freeze duration for the LUPV, LIPV, RUPV, and RIPV. Repeat cryoballoon applications to the same pulmonary vein were recorded when present.

### 2.3. Follow-Up and Definition of Recurrence

The first three-month period after the procedure was considered a blanking period. During this period, atrial arrhythmias were managed with medical therapy or cardioversion when necessary.

Arrhythmia recurrence was defined as electrocardiographically documented AF or atrial tachyarrhythmia occurring after the blanking period within one year of follow-up. Recurrence was accepted only when AF or atrial tachyarrhythmia was objectively documented by 12-lead electrocardiography or Holter monitoring. For Holter-detected episodes, a duration of ≥30 s was required. Patient-reported symptoms without electrocardiographic documentation were not counted as recurrence.

Patients were followed at 1, 3, 6, and 12 months with clinical evaluation and routine 12-lead electrocardiography. In addition to scheduled follow-up visits, recurrence could be documented on 12-lead electrocardiograms obtained during unscheduled presentations to our institution or to outside healthcare facilities for palpitations, other symptoms, or unrelated clinical reasons, when these electrocardiographic records were available in the retrospective medical record. Holter monitoring was performed when clinically indicated, particularly in patients with intermittent symptoms not captured on a standard electrocardiogram.

Continuous rhythm monitoring and universal scheduled Holter screening were not routinely performed; therefore, intermittent and asymptomatic recurrences may have been underdetected. The retrospective database did not systematically distinguish whether each documented recurrence was identified during a scheduled follow-up visit or during an unscheduled presentation, nor did it consistently record whether the patient was symptomatic at the time of electrocardiographic documentation. Consequently, the proportions of recurrences detected during scheduled versus unscheduled care and the proportions of symptomatic versus asymptomatic documented recurrences could not be determined. For the same reason, a sensitivity analysis restricted to recurrences detected during scheduled follow-up visits could not be performed.

### 2.4. Medical Therapy

Periprocedural anticoagulation followed the institutional practice in use during the study period. Direct oral anticoagulants were temporarily withheld before ablation according to the pharmacokinetic half-life of the specific agent and renal function; thus, the study did not use a fully uninterrupted DOAC strategy. In patients receiving warfarin, the international normalized ratio (INR) was assessed before the procedure and therapeutic anticoagulation was targeted within an INR range of 2.0–3.0. Oral anticoagulation was resumed after the procedure once adequate hemostasis had been achieved. All patients received oral anticoagulation for 3 months after ablation. Because the study period predated the 2024 ESC guideline update, continuation beyond 3 months followed the contemporaneous CHA_2_DS_2_-VASc-based institutional protocol. After the initial 3-month post-ablation period, anticoagulation was continued according to thromboembolic risk, defined as a CHA_2_DS_2_-VASc score of ≥1 in men and ≥2 in women. Decisions regarding long-term continuation or discontinuation were based on thromboembolic risk assessment and were not determined solely by procedural success or the absence of documented arrhythmia recurrence. The specific anticoagulant used, dose adjustments, exact duration beyond the initial 3-month period, and rates of continuation or discontinuation were not systematically recorded.

The amiodarone and propafenone variables reported in Table 1 indicate antiarrhythmic therapy initiated after the ablation procedure. These variables do not represent active antiarrhythmic drug use at the time arrhythmia recurrence was detected. Amiodarone and propafenone were not routinely continued beyond the blanking period; continuation thereafter was individualized according to clinical need.

### 2.5. Statistical Analysis

Statistical analyses were performed using IBM SPSS Statistics version 25.0 (IBM Corp., Armonk, NY, USA).

Categorical variables were expressed as frequencies and percentages and were compared using the Pearson chi-square test or Fisher’s exact test. Continuous variables were tested for normality using the Shapiro–Wilk test. Normally distributed variables were compared using the independent-samples *t*-test, whereas non-normally distributed variables were analyzed using the Mann–Whitney U test. A *p* value < 0.05 was considered statistically significant.

The primary multivariable logistic regression model was clinically specified rather than derived from univariable statistical significance or automated stepwise selection. Sex was the exposure of primary interest. Age, AF type, and left atrial diameter were included because of their established clinical relevance to recurrence after AF ablation and their potential to confound the association between sex and recurrence; these covariates were retained irrespective of their univariable *p* values. Given the limited number of outcome events, the model was intentionally kept parsimonious. Age and left atrial diameter were entered as continuous variables, whereas sex and AF type were entered as categorical variables. Male sex and paroxysmal AF were used as the reference categories. To avoid redundancy, CHA_2_DS_2_-VASc was not entered into the multivariable model because sex and age were modeled separately and the major comorbid components of the score were absent by study design. An extended sensitivity model additionally including body mass index was performed and is presented in Supplementary Table S2. Key clinical and procedural characteristics were also compared according to sex using the same distribution-appropriate tests used for the primary group comparisons and are presented in Supplementary Table S5. To evaluate potential confounding by body size, an additional sensitivity multivariable model replaced absolute left atrial diameter with left atrial diameter/BSA while retaining sex, age, and AF type; this analysis is presented in Supplementary Table S6.

Missing observations were summarized for all variables reported in the analyses (Supplementary Table S3). The primary model used complete-case analysis. The outcome, sex, age, and AF type were complete, whereas left atrial diameter was unavailable in 27 of 153 patients (17.6%); consequently, the primary model included 126 patients and 36 documented recurrence events. To explore the pattern of missing left atrial measurements, left atrial-diameter missingness was compared according to recurrence status, sex, and AF type. A full-cohort sensitivity model excluding left atrial diameter was also performed to assess whether exclusion of patients with missing left atrial measurements materially affected the sex estimate. BSA was available in 149 patients, and the combined left atrial diameter/BSA variable was available in 123 patients; therefore, the BSA-indexed sensitivity model used complete cases from these variables (n = 123).

Model assumptions and stability were examined using prespecified diagnostic analyses. Linearity of the continuous predictors in the logit was assessed using the Box–Tidwell approach. Multicollinearity was evaluated using tolerance and variance inflation factor (VIF) values. Calibration was assessed with the Hosmer–Lemeshow goodness-of-fit test and discrimination with the area under the receiver operating characteristic curve (AUC). Standardized residuals, leverage values, and Cook’s influence statistics were examined for influential observations. The primary model was additionally repeated after exclusion of the observation with the highest Cook’s influence value. Finally, 2000 simple bootstrap resamples were used to obtain bias-corrected and accelerated (BCa) 95% confidence intervals for the regression coefficients and to assess the robustness of the female-sex estimate.

### 2.6. Ethics

The study was conducted in accordance with the Declaration of Helsinki and approved by the local ethics committee (Approval number: 2025-325, Date: 03/09/2025).

## 3. Results

The study population consisted of 153 patients, including 70 women (45.8%) and 83 men (54.2%). Most patients had paroxysmal AF (n = 125, 81.7%), while persistent AF was present in 28 patients (18.3%). During the 1-year follow-up period, documented arrhythmia recurrence occurred in 40 patients (26.1%), whereas 113 patients (73.9%) had no documented recurrence. The patient-selection flow diagram details the number excluded for each predefined criterion and the final follow-up outcome status (Figure 1).

During follow-up, symptom-triggered Holter monitoring was performed in 37 patients who reported palpitations, and paroxysmal AF was documented by Holter monitoring in one patient. Documented recurrences were identified from available 12-lead electrocardiograms obtained either during scheduled follow-up or during unscheduled evaluations at our institution or outside healthcare facilities, as well as from clinically indicated Holter recordings. However, the retrospective records did not systematically identify the setting in which each of the 40 recurrences was detected or whether the patient was symptomatic at the time of documentation; therefore, recurrences could not be categorized as scheduled versus unscheduled or symptomatic versus asymptomatic events, and a scheduled-visit-only sensitivity analysis was not feasible. The frequency of symptom-triggered Holter monitoring did not differ significantly by sex (26.5% in men vs. 21.4% in women; *p* = 0.465; Supplementary Table S1), but this comparison alone cannot exclude sex-related differences in recurrence detection. Routine continuous rhythm monitoring was not performed. Regarding procedural characteristics, a second cryoballoon application in the same pulmonary vein was required in only four patients; therefore, repeat cryoballoon applications were not analyzed as a separate statistical variable.

As shown in Table 1, the proportion of female patients was significantly higher in the arrhythmia recurrence group than in the no-recurrence group (62.5% vs. 39.8%, *p* = 0.013). Post-ablation use of beta-blockers, non-dihydropyridine calcium channel blockers, amiodarone, propafenone, and digoxin did not differ significantly between the groups (all *p* > 0.05). Although persistent AF was more frequent among patients with recurrence than among those without recurrence (25.0% vs. 15.9%), the difference was not statistically significant (*p* = 0.202).

As presented in Table 2, the CHA_2_DS_2_-VASc score was significantly higher in patients with arrhythmia recurrence than in those without recurrence (median: 1.00 [IQR: 1.00] vs. 0.00 [IQR: 1.00], *p* = 0.027). No statistically significant differences were observed between the groups in age, anthropometric measurements, left atrial diameter, renal function parameters, lipid profile, inflammatory markers, hematological parameters, serum electrolytes, total protein, albumin, or CHA_2_DS_2_-VA score (all *p* > 0.05).

Procedural cryoballoon parameters were largely comparable between patients with and without AF recurrence. Among the evaluated parameters, only LIPV freeze duration showed a statistically significant difference between groups in rank distribution, despite identical median values, whereas nadir temperatures and freeze durations of the remaining pulmonary veins did not differ significantly (Table 3).

These findings suggest that the observed recurrence difference was not primarily attributable to major differences in routinely available cryoballoon procedural parameters.

The primary multivariable logistic regression was a complete-case analysis including 126 patients and 36 documented recurrence events (Table 4). Female sex was associated with documented arrhythmia recurrence (OR 2.69, 95% CI 1.14–6.37; *p* = 0.024). For left atrial diameter, the estimated OR was 1.086 per mm (95% CI 0.989–1.192; *p* = 0.085), indicating statistical uncertainty with the confidence interval compatible with little or no association as well as a potentially relevant increase in risk. Persistent AF was associated with numerically higher odds of recurrence than paroxysmal AF, but with substantial statistical uncertainty (OR 1.95, 95% CI 0.71–5.38; *p* = 0.195). Age was not associated with documented recurrence in the primary model (OR 0.990, 95% CI 0.946–1.037; *p* = 0.675). The extended model additionally including body mass index yielded similar estimates and is presented in Supplementary Table S2.

Left atrial diameter was missing in 27 patients (17.6%); among these patients, 23 had no documented recurrence and 4 had recurrence, 17 were men and 10 were women, and 21 had paroxysmal AF and 6 had persistent AF. Left atrial-diameter missingness was not significantly associated with recurrence status (*p* = 0.140), sex (*p* = 0.317), or AF type (*p* = 0.561). A complete missing-data summary for variables reported in the analyses is provided in Supplementary Table S3. In the full-cohort sensitivity model excluding left atrial diameter (n = 153; 40 recurrence events), the estimate for female sex remained similar (OR 2.72, 95% CI 1.23–5.99; *p* = 0.013).

Sex-stratified clinical and procedural characteristics are summarized in Supplementary Table S5. Compared with men, women were older (59.27 ± 10.15 vs. 53.84 ± 11.34 years; *p* = 0.002), had a lower BSA (1.876 ± 0.166 vs. 2.012 ± 0.164 m^2^; *p* < 0.001), and had a higher left atrial diameter/BSA (21.08 ± 2.81 vs. 19.21 ± 2.42 mm/m^2^; *p* < 0.001). Documented recurrence was more frequent in women than in men (35.7% vs. 18.1%; *p* = 0.013). Pulmonary vein-specific nadir temperatures and freeze durations were generally similar between sexes; LIPV freeze duration showed a difference in rank distribution despite an identical median of 240 s in both groups (*p* = 0.016). In the body-size-indexed sensitivity model, which included 123 complete cases, replacing absolute left atrial diameter with left atrial diameter/BSA attenuated the female-sex estimate (OR 2.16, 95% CI 0.90–5.18; *p* = 0.084). Left atrial diameter/BSA was also statistically uncertain (OR 1.145 per mm/m^2^, 95% CI 0.977–1.343; *p* = 0.095). Persistent AF (OR 1.98, 95% CI 0.72–5.41; *p* = 0.183) and age (OR 0.994, 95% CI 0.952–1.038; *p* = 0.790) were likewise statistically uncertain in this sensitivity model (Supplementary Table S6).

No evidence of departure from linearity in the logit was identified for age (Box–Tidwell interaction *p* = 0.672) or left atrial diameter (*p* = 0.104). Tolerance values ranged from 0.819 to 0.969 and VIF values from 1.032 to 1.220, indicating no problematic multicollinearity. The Hosmer–Lemeshow test showed no evidence of significant lack of fit (χ^2^ = 12.462, df = 8; *p* = 0.132), and model discrimination was modest (AUC 0.669, 95% CI 0.553–0.784). The maximum absolute standardized residual was 2.985, the maximum Cook’s influence statistic was 0.390, and the maximum leverage value was 0.178. Excluding the observation with the highest Cook’s influence value did not materially alter the female-sex estimate (OR 2.84, 95% CI 1.18–6.85; *p* = 0.020). Bootstrap resampling with 2000 samples likewise yielded a consistent female-sex estimate (OR 2.69, BCa 95% CI 1.13–7.26; bootstrap *p* = 0.033). Detailed model diagnostics and sensitivity analyses are summarized in Supplementary Table S4.

The frequency of symptom-triggered Holter monitoring did not differ significantly between male and female patients (26.5% vs. 21.4%, respectively; *p* = 0.465; Supplementary Table S1).

## 4. Discussion

In this highly selected low-risk cohort, female sex was associated with clinically detected, electrocardiographically documented arrhythmia recurrence under the study’s intermittent rhythm-surveillance strategy in the primary model adjusted for age, AF type, and absolute left atrial diameter. However, the female-sex estimate was attenuated and became statistically uncertain when left atrial diameter was indexed to BSA in sensitivity analysis. This pattern indicates that the primary sex association is sensitive to body-size adjustment and should not be interpreted as a definitive independent sex effect.

Arrhythmia recurrence after catheter ablation remains an important clinical problem despite technical advances and increasing procedural experience. Previous studies have reported variable recurrence rates after AF ablation, largely depending on patient characteristics, AF type, ablation strategy, and follow-up intensity [14]. In our cohort, recurrence occurred in 26.1% of patients, which is within the lower range of previously reported rates. This relatively favorable outcome may be related to the limited comorbidity burden and generally favorable clinical profile of the study population.

The association between female sex and recurrence observed in our study is consistent with previous evidence suggesting sex-related differences in AF ablation outcomes. Cheng et al. reported in a large meta-analysis that women had lower freedom from AF or atrial tachycardia recurrence after catheter ablation compared with men [15]. Similarly, a subanalysis of the FIRE AND ICE trial showed that female sex was associated with a higher risk of efficacy failure after catheter ablation for paroxysmal AF [16]. Sex-related differences in clinical presentation and ablation outcomes have also been described in the CIRCA-DOSE study [17]. In the present study, female sex was associated with documented recurrence after adjustment for age, AF type, and left atrial diameter. This finding may reflect sex-related differences in post-ablation rhythm outcomes within this cryoballoon-treated cohort, but it should not be interpreted as evidence of a direct causal effect.

Potential explanations for differences in documented recurrence between women and men include biological factors such as atrial substrate, autonomic regulation, hormonal milieu, and non-pulmonary vein triggers, as well as differences in symptom perception, referral patterns, and healthcare-seeking behavior. In this context, atrial anatomy, electrophysiological substrate, and hormonal milieu are principally sex-related biological factors, whereas symptom reporting, referral pathways, access to care, and healthcare-seeking behavior may also reflect gender-related and broader sociocultural influences. These mechanisms were not directly assessed in the present study. Menopausal status, hormone therapy, direct hormonal measurements, autonomic testing, cardiac magnetic resonance imaging, high-density electroanatomical mapping, systematic non-pulmonary vein trigger assessment, validated symptom-burden scales, and quality-of-life instruments were not available. Although the frequency of symptom-triggered Holter monitoring did not differ significantly between male and female patients, this finding does not establish equivalent recurrence ascertainment. Differences in symptom type or perception, timing of presentation, healthcare-seeking behavior, use of unscheduled care, and the likelihood of obtaining a 12-lead electrocardiogram during an intermittent event could still have influenced detection. Because the source of each documented recurrence and symptom status at the time of detection were not systematically recorded, differential ascertainment cannot be excluded. Accordingly, the observed sex association should be interpreted as an association with clinically detected, ECG-documented recurrence within this surveillance framework rather than with the total biological burden of recurrent AF. Therefore, mechanistic interpretations should be regarded as hypothesis-generating, and future prospective studies should incorporate standardized rhythm monitoring together with structural, electrophysiological, hormonal, and patient-reported outcome measures.

The deliberately restricted cohort reduced heterogeneity related to major cardiovascular and systemic comorbidities, but this design does not eliminate residual confounding and necessarily limits external generalizability. The present findings are therefore most applicable to similarly selected low-risk patients undergoing cryoballoon ablation and should not be assumed to apply to broader AF populations with hypertension, diabetes, coronary artery disease, chronic kidney disease, cerebrovascular disease, heart failure, obstructive sleep apnea, thyroid disease, or significant valvular disease. In addition, several potentially relevant covariates that may differ between women and men and may influence recurrence were not available in a sufficiently standardized or complete form for multivariable adjustment. These included AF duration and the interval from AF diagnosis to ablation; the number and duration of previous AF episodes; prior cardioversions; left atrial volume index and more comprehensive measures of atrial geometry; left ventricular ejection fraction; menopausal status and hormone therapy; alcohol use, smoking, and physical activity; validated AF-related symptom burden; antiarrhythmic drug therapy before ablation; time to pulmonary vein isolation; balloon occlusion grade; phrenic-nerve or esophageal complications; and systematic data on pulmonary-vein reconnection or repeat ablation. Consequently, the association observed for female sex may partly reflect measured and unmeasured structural, clinical, behavioral, or procedural differences rather than an independent sex effect.

The clinical meaning of AF ablation success extends beyond electrocardiographically documented rhythm recurrence. Documented recurrence, symptom recurrence, and deterioration in quality of life do not necessarily occur in parallel. Some patients may experience substantial symptomatic and functional improvement despite intermittent arrhythmia, whereas others may continue to report palpitations, fatigue, anxiety, exercise limitation, or impaired quality of life despite an apparently favorable rhythm outcome. Recent comparative data further suggest that perceived symptom relief, activity resumption, and quality-of-life improvement may differ according to ablation energy source and peri-procedural experience [18]. In addition, a recent patient-centered review emphasized that conventional electrophysiological endpoints incompletely capture post-ablation benefit and that persistent symptoms, psychological distress, unmet educational needs, healthcare utilization, and patient-reported quality of life remain clinically important even after apparently successful rhythm control [19]. In the present study, the prespecified endpoint was objectively documented arrhythmia recurrence; validated symptom-burden questionnaires, quality-of-life instruments, functional recovery, anxiety or psychological distress, patient satisfaction, and healthcare-utilization outcomes were not systematically collected. Therefore, our findings cannot determine the overall patient-perceived clinical benefit of cryoballoon ablation or whether the observed female-sex association with documented recurrence corresponds to differences in symptoms, quality of life, functional status, or healthcare use. Future prospective studies should combine standardized rhythm monitoring with validated patient-reported outcome measures.

All procedures in the present study were performed with a second-generation cryoballoon. Accordingly, the current data demonstrate an association within a cryoballoon-treated cohort but cannot establish that female sex is a technology-specific predictor or determine whether the same association would be observed with radiofrequency or pulsed-field ablation. Different ablation platforms may differ in lesion formation, procedural characteristics, recovery experience, and patient-reported outcomes. The comparative quality-of-life study cited above reported differences in perceived recovery, symptom relief, and quality of life across ablation energy sources [18], whereas a recent meta-analysis comparing pentaspline pulsed-field ablation with high-power short-duration/very-high-power short-duration radiofrequency ablation focused mainly on efficacy, safety, and procedural outcomes rather than establishing a definitive patient-reported quality-of-life advantage [20]. Therefore, cross-technology extrapolation of the present sex-related association should be avoided, and dedicated comparative prospective studies incorporating standardized rhythm surveillance and patient-reported outcomes are needed.

Routinely available cryoballoon procedural parameters were compared to explore potential procedural confounding. Pulmonary vein-specific nadir temperatures and freeze durations were broadly similar between patients with and without documented recurrence. Although LIPV freeze duration reached statistical significance in rank distribution, the median values were identical in both groups, limiting its clinical interpretability. These findings suggest that no major difference was evident in the recorded freeze-duration or nadir-temperature parameters. However, these routinely available variables do not fully characterize procedural complexity or lesion durability. Pulmonary vein anatomy and ostial dimensions, balloon occlusion grade, time to isolation, phrenic-nerve and esophageal complications, systematic assessment of pulmonary-vein reconnection, repeat-ablation findings, and non-pulmonary vein triggers were not available in a sufficiently standardized form for adjustment. Thus, unmeasured procedural or anatomical confounding cannot be excluded.

Left atrial enlargement and persistent AF are well-established factors associated with recurrence after AF ablation. Previous cryoballoon studies have identified left atrial diameter and persistent AF as important variables associated with late recurrence [21,22]. In our primary model, the estimate for absolute left atrial diameter was directionally positive but statistically uncertain (OR 1.086 per mm, 95% CI 0.989–1.192; *p* = 0.085), and persistent AF also showed higher estimated odds with a wide confidence interval (OR 1.95, 95% CI 0.71–5.38; *p* = 0.195). Because body size differs substantially between women and men, absolute anteroposterior diameter may not represent the same relative atrial dimension in both sexes. Consistent with this concern, women in the present cohort had a lower BSA but a higher left atrial diameter/BSA than men. When absolute left atrial diameter was replaced by left atrial diameter/BSA, the female-sex estimate decreased from OR 2.69 to OR 2.16 and the 95% CI included 1.0 (*p* = 0.084), while left atrial diameter/BSA itself was statistically uncertain (OR 1.145 per mm/m^2^, 95% CI 0.977–1.343; *p* = 0.095). This attenuation suggests that body-size-related differences in atrial dimensions may partly contribute to the association observed in the primary model, but it does not establish mediation or eliminate a possible sex-related effect. In addition, the BSA-indexed analysis included 123 rather than 126 patients, so the change in estimate may reflect both predictor re-expression and a small change in complete-case composition. Left atrial volume index was not available, and atrial remodeling therefore remained incompletely characterized. Taken together with the wide confidence intervals, these findings support a cautious, hypothesis-generating interpretation rather than definitive evidence of an independent causal effect of female sex.

Clinical risk scores such as CHA_2_DS_2_-VASc have previously been associated with AF recurrence after ablation [11]. In the present study, the CHA_2_DS_2_-VASc score was higher in patients with documented recurrence in the unadjusted group comparison, whereas the CHA_2_DS_2_-VA score did not differ between the groups. This finding should be interpreted with caution because female sex contributes one point to CHA_2_DS_2_-VASc and was more frequent in the recurrence group, while most of the other score components were absent or markedly restricted by the study design. Consequently, female sex and CHA_2_DS_2_-VASc were mathematically non-independent in this cohort, and interpreting the score as a separate recurrence predictor would be circular. The absence of a corresponding difference in CHA_2_DS_2_-VA further supports the interpretation that the observed CHA_2_DS_2_-VASc difference was largely driven by its sex component rather than by a greater burden of conventional thromboembolic risk factors. For this reason, CHA_2_DS_2_-VASc was not entered into the multivariable model, and its unadjusted association should not be interpreted as evidence that the score independently predicts post-ablation recurrence in this selected cohort.

Post-ablation anticoagulation was routinely maintained for 3 months in all patients. Thereafter, continuation was guided by the CHA_2_DS_2_-VASc-based protocol in use before publication of the 2024 ESC guidelines, including continuation beyond 3 months in patients with a CHA_2_DS_2_-VASc score of ≥1 in men and ≥2 in women. Importantly, long-term anticoagulation decisions were based on thromboembolic risk rather than ablation success or the apparent absence of arrhythmia recurrence. An updated systematic review and meta-analysis including 267,443 patients found that oral anticoagulation discontinuation after apparently successful AF ablation was associated with less major bleeding without a statistically significant increase in thromboembolic events; however, the authors also emphasized the need for individualized decision-making and further randomized evidence [23]. These data should therefore be interpreted as evolving evidence rather than as definitive support for routine anticoagulation withdrawal after ablation. This distinction is particularly important in the present study because the specific anticoagulant, exact treatment duration beyond 3 months, continuation or discontinuation rates, bleeding events, and thromboembolic outcomes were not systematically collected. Accordingly, our study cannot evaluate the safety or net clinical benefit of discontinuing oral anticoagulation and should not be used to guide anticoagulation withdrawal.

Although systemic inflammation has been linked to post-ablation arrhythmia recurrence, routine inflammatory markers were not associated with recurrence in our cohort [24]. The exclusion of patients with inflammatory diseases and substantial cardiovascular comorbidity may have reduced the overall inflammatory burden and attenuated the discriminatory value of these parameters. Routine inflammatory markers may therefore provide limited prognostic information in clinically homogeneous patients undergoing cryoballoon ablation.

The present findings may have clinical relevance in the context of contemporary AF management. Current guidelines increasingly support catheter ablation at earlier stages of AF and in patients with fewer accompanying diseases [1]. Consequently, recurrence predictors derived from older or more heterogeneous populations may not perform similarly in patients undergoing earlier intervention. In this cryoballoon-treated cohort, female sex was associated with documented recurrence in the primary multivariable model, but the estimate was attenuated and statistically uncertain after body-size indexing of left atrial diameter. This sensitivity to body-size adjustment, together with the limitations of rhythm surveillance and residual confounding, argues against interpreting female sex as a definitive independent predictor from the present data. Overall, the findings should be regarded as hypothesis-generating and require prospective validation in larger studies using systematic rhythm monitoring and more comprehensive assessment of atrial structure.

## 5. Conclusions

In this highly selected low-risk AF cohort treated with second-generation cryoballoon ablation, female sex was associated with higher odds of clinically detected, electrocardiographically documented arrhythmia recurrence in the primary model under an intermittent monitoring strategy based on scheduled ECGs and symptom-driven evaluations over the 1-year follow-up period. However, the female-sex estimate was attenuated and statistically uncertain when left atrial diameter was indexed to BSA, indicating that the primary association was sensitive to body-size adjustment. Because the source of each recurrence (scheduled versus unscheduled detection) and symptom status at recurrence were not systematically recorded, a sensitivity analysis restricted to scheduled-visit recurrences could not be performed. Given the retrospective design, limited number of recurrence events, non-systematic rhythm monitoring, restricted external generalizability, and potential residual confounding, these findings should be interpreted as hypothesis-generating rather than causal or definitive. Larger prospective studies using standardized rhythm monitoring and more comprehensive assessment of atrial substrate and body-size-adjusted atrial structure are needed.

### Limitations

This study has several limitations. First, the retrospective, single-center design and the modest number of documented recurrence events limit causal inference, external generalizability, and the precision of the estimated associations. The primary model was based on complete cases because left atrial diameter was unavailable in 27 patients; although missingness was not associated with recurrence status, sex, or AF type and full-cohort sensitivity analysis yielded a similar sex estimate, bias from unmeasured determinants of missingness cannot be excluded. The model was intentionally restricted to four clinically relevant variables, and despite reassuring diagnostic and bootstrap analyses, the modest event count and wide confidence intervals indicate residual risk of model instability, residual confounding, and type II error. The BSA-indexed sensitivity model included 123 complete cases and attenuated the female-sex estimate, underscoring that the primary association was sensitive to body-size adjustment; because this model also involved a slightly different complete-case sample, the source of attenuation cannot be attributed to indexing alone. Second, rhythm surveillance relied on scheduled 12-lead electrocardiography and clinically indicated Holter monitoring rather than continuous monitoring or universal scheduled ambulatory monitoring. Intermittent and asymptomatic recurrences may therefore have been missed. In addition, the retrospective records did not systematically distinguish recurrences detected during scheduled follow-up from those identified during unscheduled presentations at our institution or outside healthcare facilities, and symptom status at the time of electrocardiographic documentation was not consistently available. Thus, the relative contribution of scheduled versus unscheduled detection and symptomatic versus asymptomatic documented recurrence could not be assessed, and a sensitivity analysis restricted to recurrences identified during scheduled visits was not feasible. The similar frequency of symptom-triggered Holter monitoring in women and men does not exclude differential ascertainment, because sex- or gender-related differences in symptom perception, timing of presentation, healthcare-seeking behavior, and electrocardiographic capture of intermittent episodes may still have influenced detection. In addition, clinical success was assessed primarily through documented rhythm recurrence. Validated symptom-burden and quality-of-life instruments, functional recovery, psychological distress or anxiety, patient satisfaction, and healthcare utilization were not systematically collected; therefore, patient-perceived benefit could not be assessed and may not have paralleled electrocardiographic recurrence. Third, the highly selected low-risk cohort improves clinical homogeneity but restricts external generalizability and does not eliminate residual confounding. The results should therefore be extrapolated cautiously to patients with the major cardiovascular, metabolic, sleep-related, thyroid, or valvular disorders excluded by the study design. Several potentially important confounders were not available in a sufficiently standardized or complete form for adjustment, including AF duration and diagnosis-to-ablation interval, number and duration of previous AF episodes, prior cardioversions, left atrial volume index and more comprehensive measures of atrial geometry, left ventricular ejection fraction, menopausal status and hormone therapy, alcohol use, smoking, physical activity, validated AF-related symptom burden, pre-ablation antiarrhythmic therapy, time to pulmonary vein isolation, balloon occlusion grade, phrenic-nerve or esophageal complications, and systematic pulmonary-vein reconnection or repeat-ablation data. These unmeasured variables may differ by sex and may have contributed to the observed association. The procedural protocol also lacked a standardized dedicated phrenic-nerve monitoring strategy, and exact time-to-isolation values were not systematically stored, limiting retrospective assessment of these procedural factors. Finally, all procedures were performed with a second-generation cryoballoon, and detailed post-ablation anticoagulation exposure, continuation or discontinuation, bleeding events, and thromboembolic outcomes beyond the initial 3 months were not systematically recorded. Accordingly, the findings should not be generalized to other ablation technologies and cannot be used to infer the safety of oral anticoagulation discontinuation.

## Figures and Tables

**Table 1:** Comparison of categorical variables according to arrhythmia recurrence.

Variable	No Recurrence (n = 113)	Recurrence (n = 40)	*p* Value
Sex			0.013 a
Female	45 (39.8%)	25 (62.5%)	
Male	68 (60.2%)	15 (37.5%)	
Post-ablation beta-blocker use	33 (29.2%)	11 (27.5%)	0.838 a
Post-ablation non-DHP CCB use	17 (15.2%)	8 (20.0%)	0.480 a
Post-ablation amiodarone use	11 (9.8%)	2 (5.0%)	0.515 b
Post-ablation propafenone use	4 (3.6%)	2 (5.0%)	0.654 b
Post-ablation digoxin use	2 (1.8%)	3 (7.5%)	0.114 b
AF type			0.202 a
Paroxysmal	95 (84.1%)	30 (75.0%)	
Persistent	18 (15.9%)	10 (25.0%)	

a: Pearson’s chi-square test. b: Fisher’s exact test. Non-DHP CCB: Non-dihydropyridine calcium channel blocker.

**Table 2:** Comparison of continuous variables according to arrhythmia recurrence.

Variable	No Recurrence (n = 113)	Recurrence (n = 40)	Test Statistic	*p* Value
CRP (mg/L)	4.85 (IQR: 4.10)	5.30 (IQR: 7.28)	Z = −1.259	0.208 d
Left atrial diameter (mm)	39.00 (IQR: 3.00)	40.00 (IQR: 7.00)	Z = −1.517	0.129 d
Hemoglobin (g/dL)	12.40 (IQR: 3.30)	12.20 (IQR: 2.53)	Z = −0.557	0.578 d
Platelet count (×10^3^/µL)	250.50 (IQR: 124.50)	240.00 (IQR: 99.25)	Z = −1.246	0.213 d
White blood cell count (×10^3^/µL)	9.70 ± 2.74	9.50 ± 2.56	t = 0.415	0.679 c
Lymphocyte count (×10^3^/µL)	2.45 (IQR: 1.76)	2.71 (IQR: 1.31)	Z = −0.084	0.933 d
Monocyte count (×10^3^/µL)	0.56 (IQR: 0.56)	0.47 (IQR: 0.55)	Z = −0.578	0.563 d
Neutrophil count (×10^3^/µL)	5.86 (IQR: 3.93)	4.78 (IQR: 4.96)	Z = −0.430	0.667 d
Urea (mg/dL)	45.30 (IQR: 29.70)	50.50 (IQR: 34.80)	Z = −0.354	0.723 d
Creatinine (mg/dL)	0.94 (IQR: 0.53)	0.99 (IQR: 0.37)	Z = −1.141	0.254 d
Sodium (mmol/L)	141.00 (IQR: 10.25)	136.00 (IQR: 10.50)	Z = −1.556	0.120 d
Potassium (mmol/L)	4.60 (IQR: 1.52)	4.30 (IQR: 1.03)	Z = −1.281	0.200 d
Total cholesterol (mg/dL)	253.00 (IQR: 54.00)	238.00 (IQR: 53.50)	Z = −1.316	0.188 d
LDL cholesterol (mg/dL)	163.17 ± 35.90	152.33 ± 33.60	t = 1.579	0.117 c
Triglycerides (mg/dL)	207.65 ± 79.58	219.25 ± 87.49	t = −0.730	0.466 c
Total protein (g/L)	77.00 (IQR: 17.00)	66.50 (IQR: 12.50)	Z = −1.423	0.155 d
Albumin (g/L)	44.50 (IQR: 16.25)	42.00 (IQR: 10.75)	Z = −0.615	0.538 d
Height (cm)	170.16 ± 9.15	167.48 ± 8.48	t = 1.615	0.108 c
Body mass index (kg/m^2^)	28.20 ± 4.30	28.80 ± 5.68	t = −0.695	0.488 c
Age (years)	55.80 ± 11.25	57.83 ± 10.70	t = −0.992	0.323 c
Weight (kg)	81.44 ± 12.47	80.35 ± 13.81	t = 0.459	0.647 c
CHA_2_DS_2_-VA score	0.00 [IQR: 0.00]	0.00 [IQR: 0.00]	Z = −0.338	0.735 d
CHA_2_DS_2_-VASc score	0.00 [IQR: 1.00]	1.00 [IQR: 1.00]	Z = −2.207	0.027 d
Estimated glomerular filtration rate (mL/min/1.73 m^2^)	69.65 (IQR: 52.50)	61.30 (IQR: 48.07)	Z = −0.175	0.861 d

Normally distributed continuous variables are presented as mean ± standard deviation and were compared using the independent-samples *t*-test. Non-normally distributed continuous variables are presented as median [interquartile range (IQR)] and were compared using the Mann–Whitney U test. c: independent-samples *t*-test. d: Mann–Whitney U test.

**Table 3:** Procedural cryoballoon parameters according to arrhythmia recurrence.

Procedural Parameter	No Recurrence (n = 113)	Recurrence (n = 40)	Test Statistic	*p* Value
LUPV nadir temperature, °C	−58.0 [6.0]	−60.0 [5.0]	Z = −0.981	0.326
LIPV nadir temperature, °C	−52.0 [2.0]	−52.0 [1.5]	Z = −1.290	0.197
RUPV nadir temperature, °C	−45.0 [10.0]	−43.0 [9.5]	Z = −0.457	0.647
RIPV nadir temperature, °C	−47.0 [3.0]	−47.0 [2.75]	Z = −0.330	0.741
LUPV freeze duration, s	180.0 [90.0]	150.0 [90.0]	Z = −0.061	0.951
LIPV freeze duration, s	240.0 [0.0]	240.0 [0.0]	Z = −2.347	0.019
RUPV freeze duration, s	240.0 [0.0]	240.0 [0.0]	Z = −1.045	0.296
RIPV freeze duration, s	240.0 [0.0]	240.0 [0.0]	Z = −0.791	0.429

Data are presented as median [interquartile range]. Between-group comparisons were performed using the Mann–Whitney U test. Asymptotic two-tailed *p* values are reported. Abbreviations: IQR, interquartile range; LIPV, left inferior pulmonary vein; LUPV, left superior pulmonary vein; RIPV, right inferior pulmonary vein; RUPV, right superior pulmonary vein; s, seconds.

**Table 4:** Primary multivariable logistic regression analysis for factors associated with documented arrhythmia recurrence.

Variable	Odds Ratio (OR)	*p* Value	95% CI
Left atrial diameter (mm)	1.086	0.085	0.989–1.192
Sex (Female)	2.69	0.024	1.14–6.37
AF type (Persistent AF)	1.95	0.195	0.71–5.38
Age (years)	0.990	0.675	0.946–1.037

Complete-case analysis: n = 126, including 36 documented recurrence events. CI: confidence interval; AF: atrial fibrillation. Male sex and paroxysmal AF were used as the reference categories.

## Data Availability

The data presented in this study are available from the corresponding author upon reasonable request. The data are not publicly available due to privacy and ethical restrictions.

## Supplementary Materials

The following supporting information can be downloaded at: https://www.mdpi.com/article/10.3390/jcm15166448/s1, Table S1. Frequency of symptom-triggered Holter monitoring according to sex; Table S2. Extended sensitivity multivariable logistic regression model for documented arrhythmia recurrence; Table S3. Missing-data summary for variables reported in the analyses; Table S4. Primary-model diagnostics and sensitivity analyses; Table S5. Key clinical and procedural characteristics according to sex; Table S6. Sensitivity multivariable logistic regression model using body surface area-indexed left atrial diameter.
