# Supplementary material for: Factors Associated with Documented Arrhythmia Recurrence After Cryoballoon Ablation in a Low-Risk Atrial Fibrillation Population Without Major Comorbidities
Source: J Clin Med. 2026 Aug 20;15(16):6448. doi: 10.3390/jcm15166448 (PMC13513720; doi:10.3390/jcm15166448)
Supplement: Supplementary file 1 [file jcm-15-06448-s001.zip › jcm-4461246-supplementary.pdf]

Supplementary Materials

Supplementary Table S1. Frequency of symptom-triggered Holter monitoring according to sex.

Variable	Male (n = 83)	Female (n = 70)	p value
Holter monitoring			0.465
No	61 (73.5%)	55 (78.6%)	
Yes	22 (26.5%)	15 (21.4%)	

Data are presented as number and percentage. Between-group comparison was performed using Pearson's chi-square test.

Supplementary Table S2. Extended sensitivity multivariable logistic regression model for documented arrhythmia recurrence.

The extended sensitivity model additionally included body mass index in addition to the four clinically selected variables used in the primary multivariable model.

Variable	Odds Ratio (OR)	p value	95% CI
Left atrial diameter (mm)	1.087	0.080	0.990–1.193
Sex (Female)	2.78	0.022	1.16–6.62
AF type (Persistent AF)	1.96	0.197	0.70–5.43
Age (years)	0.994	0.805	0.950–1.041
Body mass index (kg/m^2)	0.969	0.486	0.886–1.059

CI: confidence interval; AF: atrial fibrillation. Male sex and paroxysmal AF were used as the reference categories.

Supplementary Table S3. Missing-data summary for variables reported in the analyses.

Variable	Valid n	Missing n	Missing (%)
Documented recurrence outcome	153	0	0.0
Sex	153	0	0.0
AF type	153	0	0.0
Post-ablation beta-blocker use	153	0	0.0
Post-ablation non-DHP CCB use	152	1	0.7
Post-ablation amiodarone use	152	1	0.7
Post-ablation propafenone use	152	1	0.7
Post-ablation digoxin use	152	1	0.7
CRP	145	8	5.2
Left atrial diameter	126	27	17.6
Hemoglobin	151	2	1.3
Platelet count	151	2	1.3
White blood cell count	151	2	1.3
Lymphocyte count	151	2	1.3
Monocyte count	151	2	1.3
Neutrophil count	151	2	1.3
Urea	151	2	1.3
Creatinine	151	2	1.3
Sodium	151	2	1.3
Potassium	150	3	2.0
Total cholesterol	137	16	10.5
LDL cholesterol	136	17	11.1
Triglycerides	136	17	11.1
Total protein	130	23	15.0
Albumin	110	43	28.1
Height	149	4	2.6
Body mass index	149	4	2.6
Body surface area	149	4	2.6
Left atrial diameter/BSA	123	30	19.6
Age	153	0	0.0
Weight	149	4	2.6
CHA_2DS_2-VA score	153	0	0.0
CHA_2DS_2-VASc score	153	0	0.0

Variable	Valid n	Missing n	Missing (%)
Estimated glomerular filtration rate	151	2	1.3
LUPV nadir temperature	151	2	1.3
LIPV nadir temperature	151	2	1.3
RUPV nadir temperature	149	4	2.6
RIPV nadir temperature	147	6	3.9
LUPV freeze duration	151	2	1.3
LIPV freeze duration	151	2	1.3
RUPV freeze duration	149	4	2.6
RIPV freeze duration	147	6	3.9
Repeat cryoballoon application to the same pulmonary vein	153	0	0.0

For the primary multivariable model, the outcome, sex, age, and AF type were complete; missingness was entirely attributable to left atrial diameter (27/153, 17.6%), resulting in a complete-case sample of 126 patients with 36 recurrence events. Among patients with missing left atrial diameter, 23 had no documented recurrence and 4 had recurrence; 17 were men and 10 were women; and 21 had paroxysmal AF and 6 had persistent AF. Left atrial-diameter missingness was not significantly associated with recurrence status ($p = 0.140$), sex ($p = 0.317$), or AF type ($p = 0.561$).

Supplementary Table S4. Primary-model diagnostics and sensitivity analyses

Diagnostic/sensitivity analysis	Result
Primary complete-case sample	n = 126; 36 recurrence events
Linearity in logit: age × ln(age)	$p = 0.672$
Linearity in logit: LA diameter × ln(LA diameter)	$p = 0.104$
Tolerance	0.819–0.969
Variance inflation factor	1.032–1.220
Hosmer–Lemeshow goodness-of-fit	$\chi^2 = 12.462$; df = 8; $p = 0.132$
Discrimination	AUC = 0.669; 95% CI 0.553–0.784
Maximum absolute standardized residual	2.985
Maximum Cook's influence statistic	0.390
Maximum leverage	0.178
Highest-Cook's-case exclusion sensitivity	Female sex OR 2.84; 95% CI 1.18–6.85; $p = 0.020$
Full-cohort sensitivity model excluding LA diameter	n = 153; female sex OR 2.72; 95% CI 1.23–5.99; $p = 0.013$
Bootstrap resampling	2,000 samples; female sex OR 2.69; BCa 95% CI 1.13–7.26; $p = 0.033$

AUC: area under the receiver operating characteristic curve; BCa: bias-corrected and accelerated confidence interval; CI: confidence interval; LA: left atrial; OR: odds ratio.

Supplementary Table S5. Key clinical and procedural characteristics according to sex.

Variable	Male (n = 83)	Female (n = 70)	p value
Age (years)	53.84 ± 11.34	59.27 ± 10.15	0.002
Height (cm)	174.96 ± 6.94	163.03 ± 6.62	<0.001
Weight (kg)	83.60 ± 11.83	78.30 ± 13.38	0.011
Body mass index (kg/m²)	27.29 ± 3.42	29.59 ± 5.61	0.004
Body surface area (m²)	2.012 ± 0.164	1.876 ± 0.166	<0.001
Left atrial diameter/BSA (mm/m²)	19.21 ± 2.42	21.08 ± 2.81	<0.001
Estimated glomerular filtration rate (mL/min/1.73 m²)	83.20 [48.53]	57.00 [35.90]	<0.001
CHA_2DS_2-VA score	0.00 [0.00]	0.00 [1.00]	0.056
AF type			0.110
Paroxysmal AF	64 (77.1%)	61 (87.1%)	
Persistent AF	19 (22.9%)	9 (12.9%)	
Documented arrhythmia recurrence	15 (18.1%)	25 (35.7%)	0.013
LUPV nadir temperature (°C)	−60.0 [6.0]	−60.0 [6.0]	0.915
LIPV nadir temperature (°C)	−52.0 [0.0]	−52.0 [1.0]	0.869
RUPV nadir temperature (°C)	−45.0 [10.0]	−44.0 [8.0]	0.482
RIPV nadir temperature (°C)	−47.0 [2.0]	−47.0 [3.0]	0.980
LUPV freeze duration (s)	180.0 [90.0]	150.0 [90.0]	0.990
LIPV freeze duration (s)	240.0 [0.0]	240.0 [0.0]	0.016
RUPV freeze duration (s)	240.0 [0.0]	240.0 [60.0]	0.336
RIPV freeze duration (s)	240.0 [0.0]	240.0 [0.0]	0.427
Repeat cryoballoon application to the same pulmonary vein	1 (1.2%)	3 (4.3%)	0.333

Normally distributed continuous variables are presented as mean ± standard deviation and were compared using the independent-samples t-test; for body mass index, the Welch t-test was used because of unequal variances. Non-normally distributed continuous variables are presented as median [interquartile range] and were compared using the Mann–Whitney U test. Categorical variables are presented as number (percentage) and were compared using Pearson's chi-square test or Fisher's exact test, as appropriate. Variable-specific valid sample sizes differed because of missing data: LA diameter/BSA, 64 men and 59 women; eGFR, 82 men and 69 women; LUPV/LIPV temperature and freeze duration, 83 men and 68 women; RUPV temperature and freeze duration, 82 men and 67 women; RIPV temperature and freeze duration, 81 men and 66 women. BSA: body surface area; AF: atrial fibrillation; LUPV: left superior pulmonary vein; LIPV: left inferior pulmonary vein; RUPV: right superior pulmonary vein; RIPV: right inferior pulmonary vein.

Supplementary Table S6. Sensitivity multivariable logistic regression model using body surface area-indexed left atrial diameter.

Variable	Odds Ratio (OR)	p value	95% CI
AF type (Persistent AF)	1.98	0.183	0.72–5.41
Sex (Female)	2.16	0.084	0.90–5.18
Age (years)	0.994	0.790	0.952–1.038
Left atrial diameter/BSA (mm/m^2)	1.145	0.095	0.977–1.343

Complete-case sensitivity analysis: n = 123. Absolute left atrial diameter in the primary model was replaced by left atrial diameter indexed to body surface area. Male sex and paroxysmal AF are presented as the reference categories to maintain consistency with the primary manuscript model. In this body-size-adjusted sensitivity analysis, the female-sex estimate was attenuated and statistically uncertain (OR 2.16, 95% CI 0.90–5.18; p = 0.084). BSA: body surface area; AF: atrial fibrillation; CI: confidence interval; OR: odds ratio.
